# Design Aspects for Portable LED-Based Colorimetric Characterisation Systems Targeting Liquid Analytes

**DOI:** 10.3390/s24061960

**Published:** 2024-03-19

**Authors:** François Dupont, Serguei Stoukatch, Philippe Laurent, Kasper Eersels, Bart van Grinsven, Jean-Michel Redouté

**Affiliations:** 1 Microsys Laboratory, Department of Electrical Engineering and Computer Science, University of Liège, 4000 Liège, Belgium; 2 Sensor Engineering Department, Faculty of Science and Engineering, Maastricht University, 6200 MD Maastricht, The Netherlands

**Keywords:** colorimetric characterisation, chemical analysis, optical sensor, RGB sensors

## Abstract

Colorimetric characterisation systems based on LEDs and RBG sensors are straightforward to implement, are highly integrable allowing for portable measurement systems and can be constructed using widespread and affordable components. They have already proved to be a satisfactory solution in several applications related to chemical analysis. In this paper, we present an RGB sensor-based prototype for colorimetric characterisation, which can accommodate cuvettes with optical paths of 10 mm and 40 mm. We assessed the impact of experimental condition parameters such as the variability of the analyte volume in the cuvette, as well as the presence of floating particles or deposits at the bottom of the cuvette. While these would not impact the result given by a spectrophotometer that generally has a directional light source, they must be considered in LED/RGB sensor analysers in which the light path is not tightly controlled. We demonstrated that there is a minimal sensor height above the bottom of the cuvette and a minimal analyte level (both depending on the prototype optical path length) above which the analyte volume and the presence of floating particles and deposits have no impact on the prototype output signal. Finally, based on these results, we proposed a test method for a quick dye-displacement assay, in which the reagent is a dye-loaded molecularly imprinted polymer that is poured directly into a cuvette.

## 1. Introduction

Colorimetric characterisation methods date back to the 19th century and are constantly evolving. When they relate to chemical analysis, the basic principle of these methods is to detect the occurrence of a specific chemical reaction, which translates into a colour change of the analyte. The detection of colour variations also has various alternative applications, for example, for the quality assessment and ageing detection of a variety of fluids, from petrochemical products to pharmaceutical solutions. A large panel of colorimetric measurement technologies, whether based on spectrophotometer devices, camera image acquisition, scanners or RGB sensor systems, have been extensively described in the scientific literature [1]. Traditional spectrophotometers, on the one hand, are usually expensive and bulky instruments suited for use in a laboratory. Such instruments include a monochromator that splits the light source into individual spectral components, which allows for the targeting of one or several specific bandwidth(s) of interest for the application. Recent works include the design of more portable and lower-cost spectrophotometer devices. In [2], the authors present a smartphone-based multi-channel spectrophotometer. This prototype was demonstrated for the pH measurement of water. In [3], the design and implementation of a microcontroller-based spectrophotometer are presented. This was validated for the detection of mercuric ions in water.

Single-pixel LED-based systems, on the other hand, do not involve any complex optical elements and can usually be implemented with a handful of low-cost and miniature off-the-shelf electronic components. They are therefore amongst the most cost-effective and portable colorimetric characterisation systems. While such devices do not aim to compete with spectrophotometers in terms of frequency resolution, recent studies have reported that they are usable for quantitative colorimetric characterisation and can achieve, in several cases, analytical performance similar to that obtained with commercial spectrophotometers [4]. Depending on the target application, the light source can be either a coloured LED, whose peak frequency matches the absorption frequency of the analyte, or a white LED, allowing for more versatility. In a similar fashion, the sensor can be either a single photodiode or an RGB sensor. An RGB sensor is usually composed of a triplet of photodiodes, each sensitive in a different frequency range, allowing for a complete coverage of the visible spectrum. Therefore, compared to a single photodiode, RGB sensors are able to identify in which frequency range the observed phenomenon occurs. In [5], the authors present an RGB sensor-based system for the monitoring of nitrite in aquaculture ponds. Peristaltic pumps were used to automatically sample and mix pond water with a reagent in a detection chamber in which the RGB sensor was located. A white LED was used as the light source. In [6], an alternative apparatus for nitrite determination in water, based on Griess’ method, is presented. A green LED was used as the light source, and the green channel of an RGB sensor was selected as the output signal. The measurement was performed in a semi-micro-polystyrene cuvette, and the process is described as being environmentally friendly, as it requires a very low reagent volume. Nitrite monitoring is also of interest for health-related applications. In [7], a portable and low-cost UV-based LED system is presented for the detection of nitrite in urine, allowing for the diagnosis of bacterial infection. In this case, a single Si-pin photodiode is used as the sensor. In [8], the authors describe a system intended to characterise the pH of a solution. In this work, the analyte is mixed in a cuvette with several reagents that undergo colour changes in different pH ranges. A white LED is used to illuminate the sample, and the three channels of an RGB sensor are used to obtain a quantitative evaluation of the analyte’s pH. This enables pH measurement over the whole pH range, compared to colorimetric pH measurement systems based on a single reagent and a single photodiode [9].

In addition to the numerous applications based on the detection of a colour change of a solution as a chemical reaction occurs between an analyte and a reagent, colorimetric measurement systems using LEDs have also been used for alternative applications. In [10], an RGB sensor was used in tandem with an IR-based turbidimeter in order to determine the colour of suspended particles in water. The device was placed on the structure of a buoy and tested under real conditions for two months. In [11], a near-infrared LED photometer dedicated to the analysis of gasoline is presented. Thanks to its portability, it allows for field analyses to be carried out. Finally, we believe that several applications will emerge in the pharmaceutical field, as the colour variation of liquid drugs indicates the presence of impurities or of product degradation, and these tests can be carried out rapidly at a minimal cost [12].

In this paper, we first present an RGB sensor-based prototype for liquid analytes’ colorimetric characterisation. While some works report using a custom fluidic cell [13], we decided to use standard commercially available cuvettes as the analyte container. Using these cuvettes speeds up the development of a measurement system (e.g., using a 3D printer to build the cuvette holder). They ensure repeatability, and they can be easily swapped or cleaned between tests, while the cuvette holder and optoelectronic systems can be reused indefinitely. We developed two versions of the prototype, allowing for the accommodation of cuvettes with optical paths (OPs) of 10 mm and 40 mm. The 10 mm OP cuvettes are the most standard, while the 40 mm OP cuvettes should allow for increasing the system’s sensitivity when there is less constraint on the analyte volume or on the system dimensions.

Subsequently, we assess the impact of the analyte volume/height in the cuvette on the signal output. As the design of the prototype is straightforward, the light path is not as strictly controlled as it would be when using a laboratory spectrophotometer. Therefore, we expected the light reflection at the analyte/air interface to play a significant role in the output signal variation, at least for low analyte volumes. The objectives of this study were twofold. First, LED-based systems, owing to their portability, simplicity and cost-effectiveness, will find many applications in measurements performed in the field, outside of a controlled laboratory environment. In such environments, strict measurement protocols are more difficult to follow, especially if the measurements are performed by non-specifically trained people. Although the use of portable laboratory equipment, such as high-accuracy pipettes, would facilitate the process of volume control, these remain fragile and expensive. Therefore, we expect that the analyte level will often be inaccurately controlled in such circumstances. Second, for applications taking place in the laboratory, even if the volume can easily be controlled (and certainly must be in the case where an analyte and a reagent are mixed), there is often an economical and/or environmental driver to minimise the quantity of the analyte and reagent to be used to perform one test. In this study, we experimented with both water and dye solutions.

Finally, building upon previously presented results, we introduce a new measurement concept for LED-based system, in which the reagent is in the solid phase. For the preliminary experiments presented in this paper, we used a dye-loaded molecularly imprinted polymer (MIP), taking the form of particles with an average diameter of 100 µm. As particles floating on the analyte surface are difficult to avoid in this case, we studied how these particles, as well as deposits at the bottom of the cuvette, affect the measurements. The objective of these dye-displacement assays was indeed to measure the amount of dye released in the solution, which is an image of the concentration of the MIP target in the analyte, but the result should be insensitive to the unreleased MIP particles.

The structure of this paper is as follows: The prototype hardware is described in Section 2. Section 3 presents the results related to the analyte volume variation and to the impact of deposits and floating particles. Finally, Section 4 draws the conclusions.

## 2. Materials and Methods

**Figure 1:**
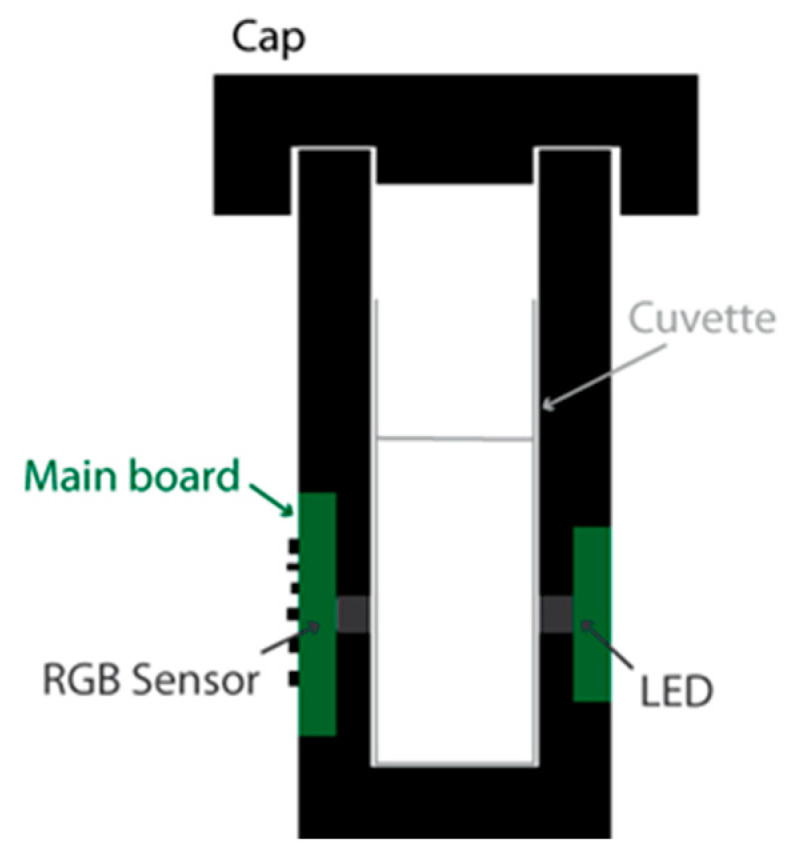
Prototype cross-sectional schematic view.

The prototype was designed so as to be suitable for as many applications as possible. For this purpose, we selected a white LED as the light source and an RGB sensor as the transducer. Generally speaking, two major LED/RGB sensor implementations have been reported. In the first implementation [4,5], the LED and the sensor are placed next to each other on the same PCB (Printed Circuit Board), and the light reflects on a surface on the other side of the cuvette. This implementation is more common in the literature, likely because it can be implemented using RGB sensor development kits, which usually come in this configuration. Another advantage is that, because the light reflects on the white enclosure wall and reaches the sensor after a round trip in the analyte, the sensitivity is likely increased, as the optical attenuation is directly proportional to the optical path length, as expressed by the Beer–Lambert law. However, the sensor output depends on the reflectivity of the enclosure material (composition, colour and surface finish), and the effective light paths from the LED to the sensor are less straightforward to estimate. For these reasons, we decided to implement an alternative configuration (Figure 1) in which the RGB sensor and the LED are located on the facing sides of the cuvette [6]. An opaque enclosure, including a cap, was implemented to eliminate stray light inside the system. This enclosure was 3D printed using black PLA filament. In the first implementation of the prototype, the LED and the sensor are placed precisely to face each other, 7.5 mm above the enclosure bottom and 6.5 mm above the cuvette bottom (taking into account a 1 mm cuvette wall thickness). For the experiments presented in Section 4, we also used a modified version of the prototype in which the LED/RGB sensor centre line is located higher, i.e., 14 mm above the bottom of the cuvette.

**Figure 2:**
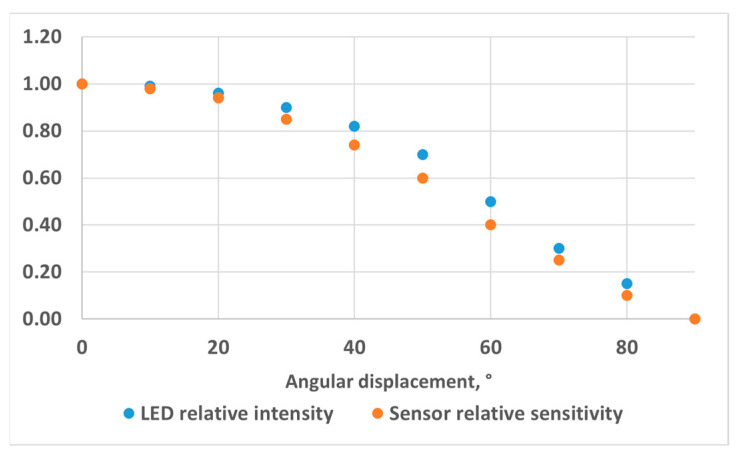
LED relative intensity and RGB sensor relative sensitivity as a function of the displacement angle.

As the main component of our colorimetric measurement system, we selected the VEML3328 RGB sensor from Vishay (Malvern, PA, USA). This is a multi-use colour sensor with red, green, blue, clear and infrared (IR) channels, with a 16-bit resolution for each channel. The interface circuit is based on the development kits SensorXplorer and VEML3328-SB from Vishay (Malvern, PA, USA). We fully redesigned the electronic hardware layout while keeping the electronic schematic equivalent. In this way, the software libraries provided by the manufacturer to configure the sensor and record the measurements are still compatible, and we used the software (VEML3328_Xplorer_v1.4.1) provided with the development kits to configure the sensor and record data. The updated hardware consists of two PCBs. The main PCB, connected to a PC through a USB cable, includes most of the electronic components on the top layer. The only component on the bottom layer is the VEML3328 sensor, which faces towards the cuvette. The second PCB is populated with a standard 400 to 800 nm white LED with a 5500 K colour temperature (reference VLMW33S2V1-5K8L-08, from Vishay (Malvern, PA, USA). The two boards are connected using an FFC (Flat Flexible Cable) Premo-Flex 15266-0053 from Molex (Lisle, IL, USA). The LED is powered by a voltage of 5 V through a resistor of 47 Ω, leading to a typical LED current of 27.7 mA (considering a typical LED forward voltage of 3.7 V). The relative intensity of the LED [14] and the relative sensitivity of the RGB sensors [15] are depicted in Figure 2 as a function of angular displacement. The angle of half intensity of the LED is about 60°. The sensor gain was set to 1, while the integration time was set to 50 ms for 10 mm OP cuvettes and to 200 ms for 40 mm OP cuvettes. This is the largest integration time that we could use through experiments without saturating the sensor output.

**Figure 3:**
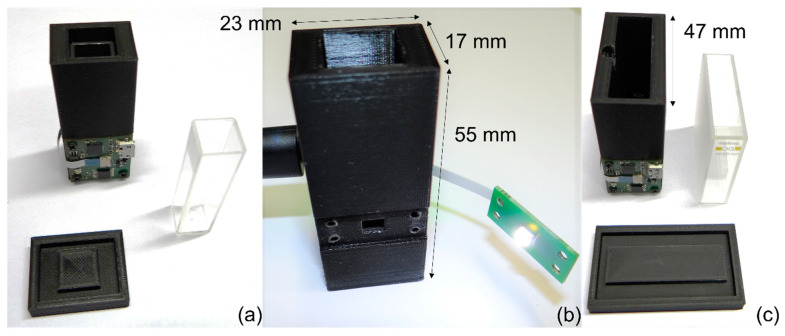
Prototype overview: (**a**) frontside with the main PCB board, cuvette and cap, 10 mm OP; (**b**) backside with unmounted LED PCB, 10 mm OP; (**c**) frontside with the main PCB board, cuvette and cap, 40 mm OP.

The final prototype implementation is shown in Figure 3. The primary setup uses a disposable plastic cuvette, which has an optical path of 10 mm (VWR 612-5503). In an alternative implementation, we used a larger cuvette with an optical path of 40 mm. As plastic cuvettes of the latter dimension were not easily available, we used an optical glass cuvette 6030-OG from Hellma Analytics (Müllheim, Germany) that was carefully cleaned between each test. This alternative prototype is very similar, with the only difference being that the 3D printed enclosure is made much larger to accommodate the 40 mm glass cuvette (a width of 47 mm instead of 17 mm).

**Figure 4:**
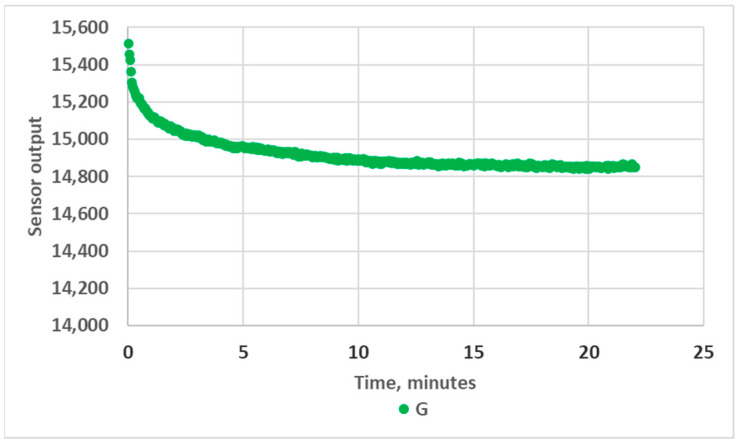
Variation in RGB sensor readings during warm-up, green channel, empty 10 mm OP prototype.

It should be noted that a warm-up period of 10 to 15 min was necessary to obtain stable measurements. This likely originated from the significant heating of the LED, as no specific heat sink was implemented. The LED power dissipation was indeed about 100 mW (V_F_ = 3.7 V, I_F_ = 27.7 mA), leading to an LED junction maximum temperature increase of about 40 °C (R_thJA_ = 400 K/W). This effect, illustrated in Figure 4, must be taken into account if the prototype is intended for quick analyses in the field.

**Figure 5:**
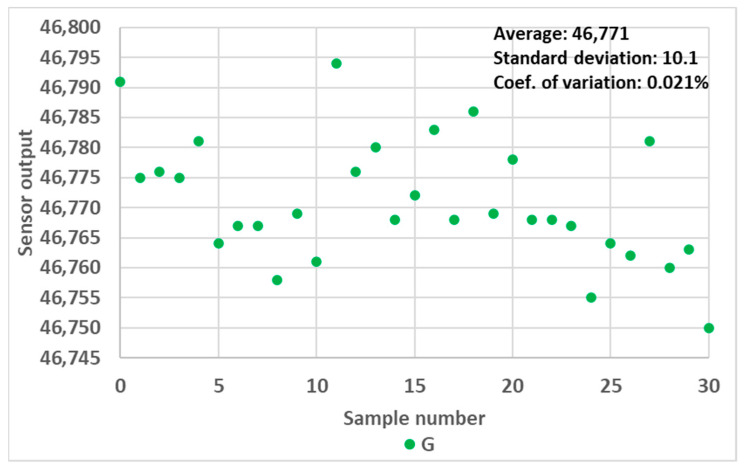
Variation in RGB sensor readings over 30 consecutive measurements, green channel, 10 mm OP prototype filled with 0.9 mL water.

Finally, each measurement point presented from here onwards represents the average value over 30 successive sensor readings. The sensor readings proved to be very stable over the successive readings, as illustrated for one random case in Figure 5. The coefficient of variation of the readings never exceeded 0.1% during the experimentation. Therefore, we omitted the error bar in subsequent figures for the sake of clarity.

## 3. Results

### 3.1. Impact of the Analyte Level in the Cuvette

**Figure 6:**
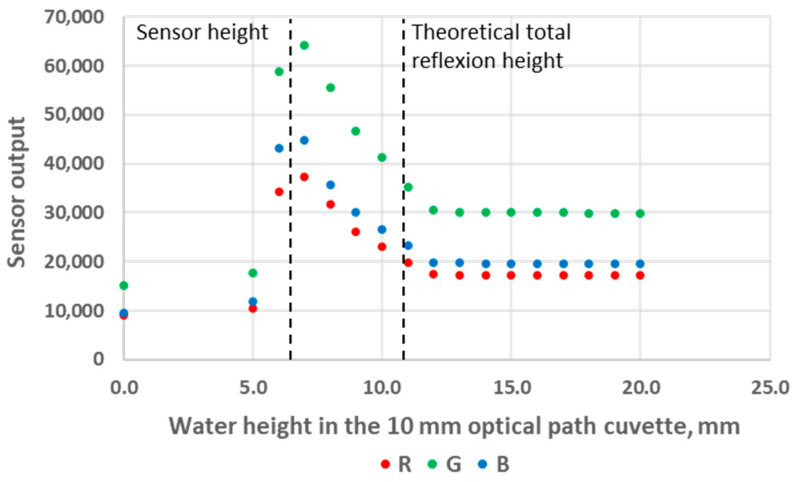
Sensor output variations as a function of water level height in the 10 mm OP cuvette.

**Figure 7:**
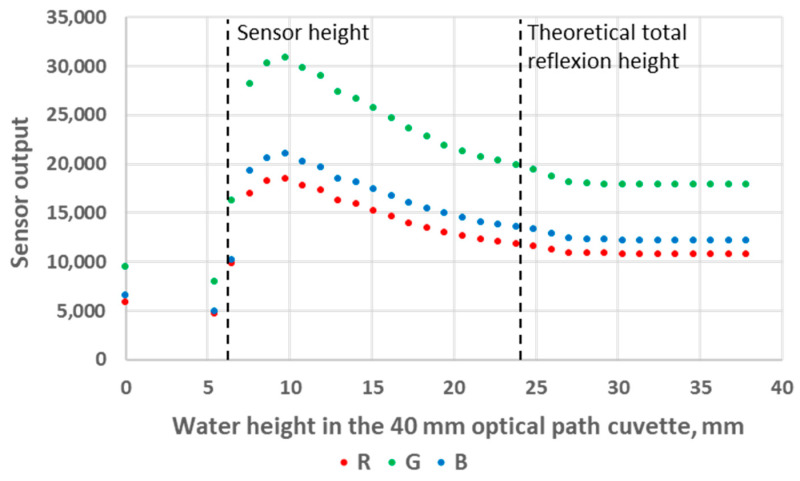
Sensor output variations as a function of water level height in the 40 mm OP cuvette.

We first used standard deionised water as the analyte, and we experimented with both 10 mm and 40 mm OP cuvettes. The results are presented in Figure 6 and Figure 7. These figures can be divided into three areas. The first area corresponds to analyte heights below the LED/sensor level. This area does not relate to an intended use case of the system and was not studied further in this work. The second area starts as the water level reaches the LED/sensor level. At this point, the sensor output signal rises abruptly until reaching a maximum value and subsequently starts to decrease. Finally, in the third area, the sensor output signal stabilises and no longer exhibits any variation with respect to the water level. The fact that the output signal of the sensor is higher in this third area compared to the first area can be explained as follows: Disregarding any reflection, while the light beams transition between the plastic of the cuvette to a less refractive material (air or water), the refraction angle increases the most in the least refractive medium. Consequently, less energy directly reaches the RGB sensor in air compared to in water. It should be noted that there is an air gap between the LED/sensor surface and the plastic cuvette and that additional optical phenomena take place at these interfaces. However, this remains constant in all experiments and will not be considered. From the inner surface of the cuvette, the LED and the sensor cannot be considered punctual elements, not only because of the intrinsic geometrical dimensions of their constituents but also because of the aforementioned reflections and refractions that occur in the component/air/plastic light path. This explains why the maximum of the sensor output is not reached immediately as the water height reaches the LED/sensor centre level but rather happens slightly above this level.

**Figure 8:**
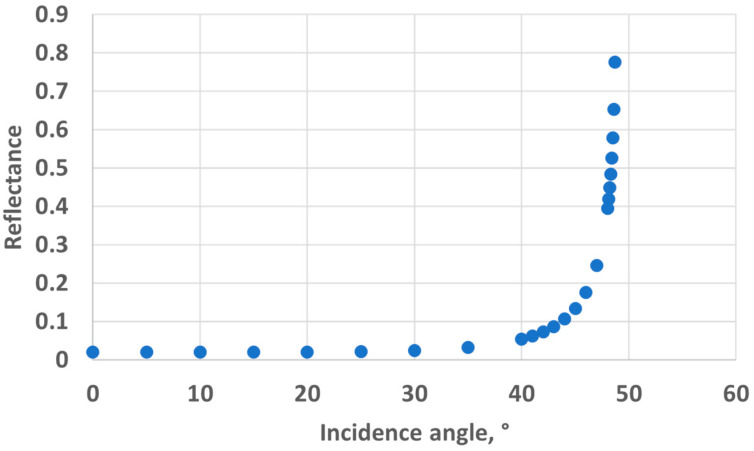
Reflectance at the water-to-air interface.

**Figure 9:**
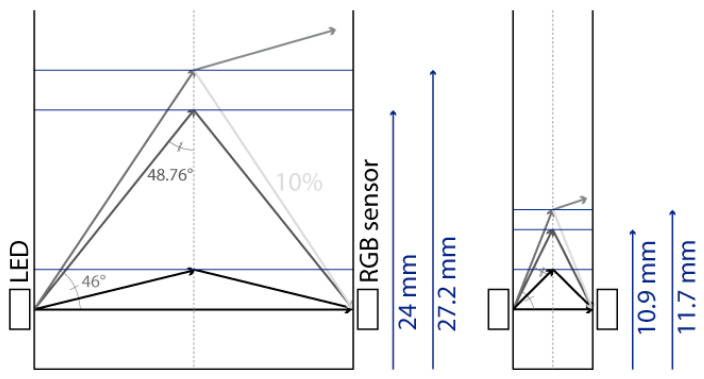
Schematic representation of light reflection and/or refraction at different water-to-air incidence angles.

The main observation from these experiments is that there is a water height above which the sensor signal output stabilises. To understand the reason for this, we first calculated the theoretical water height corresponding to the total reflexion angle. Considering that the refractive index of water is *n*_1_ = 1.33, the refractive index of air is *n*_2_ = 1, and Snell’s law *n*_1_ *sin θ*_1_ *= n*_2_ *sin θ*_2_ (where *θ*_1_ and *θ*_2_ are the incidence angle and the refraction angle, respectively), the value of *θ*_1_ corresponding to a refraction angle of *θ*_2_ = 90° is *θ*_1_ = 48.76°. In the case of the 10 mm OP cuvette, this corresponds to a water level of 4.4 mm above the LED/sensor level (10.9 mm above the bottom of the cuvette). In the case of the 40 mm OP cuvette, this corresponds to a water level of 17.5 mm above the LED/sensor level (24 mm above the bottom of the cuvette). The experimentally obtained threshold value is, hence, in good agreement with this theoretical prediction, though slightly higher: about 1 mm for the 10 mm OP cuvette and about 4 mm for the 40 mm OP cuvette. Above the water level corresponding to the total reflection limit, the reflectance, i.e., the percentage of energy reflected at the surface, quickly decreases. The reflectance values with respect to the incidence angle are presented in Figure 8 and were calculated from Fresnel Equations (1) and (2), considering the average value of s-polarised and p-polarised components, as the LED light is unpolarised [16]. As can be seen in Figure 8, the reflectance drops below 10% at an incidence angle equalling 44°. This represents an extra height close to 0.8 mm and 3.2 mm for the prototypes with OPs of 10 mm and 40 mm, respectively. This result is in good agreement with the measurements presented in Figure 6 and Figure 7. Above this extra height, the amount of reflected energy becomes very low, which, combined with a decreased relative intensity of the LED (about 70% at 46°) and a decreased RGB sensor relative sensitivity (about 60% at 46°), leads to a stabilised output signal, as schematised in Figure 9.

(1) $$R_{s}={\left|\frac{n_{1}\cos\theta_{1}-n_{2}\cos\theta_{2}}{n_{1}\cos\theta_{1}+n_{2}\cos\theta_{2}}\right|}^{2}$$

(2) $$R_{p}={\left|\frac{n_{1}\cos\theta_{2}-n_{2}\cos\theta_{1}}{n_{1}\cos\theta_{2}+n_{2}\cos\theta_{1}}\right|}^{2}$$

The sensor output decreases in the middle parts of Figure 6 and Figure 7 also originate from a decrease in the reflected light energy contribution in the sensor signal linked to the decrease (resp. increase) in the LED (resp. sensor) relative intensity (resp. sensibility) with the increase in the optoelectronic angular displacement. The increased energy absorption that occurs in the water as the optical path of the reflected beam increases with the water level, according to the Beer–Lambert law, is unlikely to be predominant. Indeed, the absorption coefficient of visible light in water is low and varies from 0.011 m^−1^ at 470 nm (peak sensitivity wavelength of the RGB sensor’s blue channel) to 0.264 m^−1^ at 610 nm (peak sensitivity wavelength of the RGB sensor’s red channel), so R, G and B curves would present visibly different behaviour if absorption in water was significant [17].

**Figure 10:**
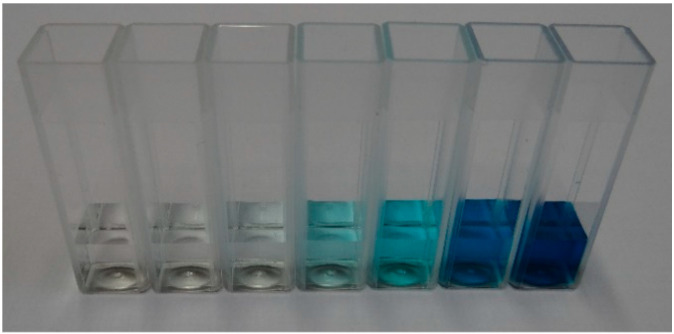
Malachite green solution, 1 mL; 0 mM to 0.1 mM.

In the second phase, we proceeded with the prototype characterisation using dye-loaded solutions, and we focused on 10 cm OP cuvettes. We used two dyes, namely, crystal violet (CV) and malachite green (MG). MG samples were prepared on the same day as the experiments took place, as this dye is known for its low stability [18]. CV samples were prepared the day before testing, as this dye is more stable [19]. Each dye was prepared in several concentrations: 0, 0.0005, 0.001, 0.005, 0.01, 0.05 and 0.1 mM. We selected two analyte heights of 10 mm (Figure 10) and 25 mm, for which the impact of analyte volume accuracy, based on previous results, was expected to be high and insignificant, respectively. We used a standard syringe to pour the analytes in the cuvette, hereby mimicking an approximate and unquantified control of the volume repeatability for each dye concentration, which would typically be encountered in the field. According to [20], we anticipated that the error in the volume could be higher than 10%, with this error increasing with the ratio of the syringe volume to the volume of analyte used.

**Figure 11:**
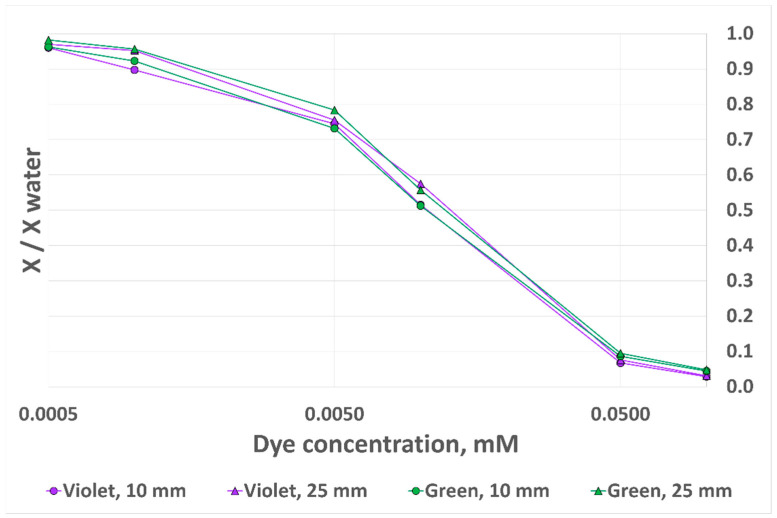
RGB sensor output signal variation with dye concentration for two dyes and two analyte heights, 10 mm OP prototype.

We first considered one component of the RGB triplet, according to the dye absorption spectrum: green (G) for CV and red (R) for MG. The output metrics of this experiment, X/X_water_, correspond to the sensor output signal divided by the reference signal obtained with clear water. The results for the four different test configurations are presented in Figure 11 (“X” on the Y-axis stands for G in the case of CV and for R in the case of MG in order to fit both measurements on the same plot). If the analyte level was rigorously similar between experiments, and assuming identical experimental conditions, we would expect the X/X_water_ output signal variation with dye concentration to be identical whether the analyte level was 10 mm or 25 mm. However, as can be observed in Figure 11, there are several discrepancies between the triangle-marked measurements (2.5 mL/25 mm), which are considered the reference measurements, as we showed that analyte volume accuracy has no impact at this height, and the circle-marked measurements (1 mL/10 mm). The X/X_water_ output signal error reaches 9.8% for MG and 12.8% for CV.

We observed previously that, although RGB sensor outputs are sensitive to analyte heights in a certain range, each R, G and B component varies in a similar manner. For this reason, we decided to experiment with alternative output metrics. We considered not only the component of interest (G for CV and R for MG) but also its relative proportion in the R/G/B triplet. It was therefore expected that an increase (resp. a decrease) in a single output channel (due to analyte volume inaccurate control) would be, at least partially, compensated by the overall increase (resp. decrease) in the sum of the three channels. For this, we defined a new variable as follows:

(3) $$Xr=\frac{X}{R+G+B},\;\mathrm{with}\;X=G\;\mathrm{for}\;\mathrm{CV}\;\mathrm{and}\;X=R\;\mathrm{for}\;\mathrm{MG}.$$

**Figure 12:**
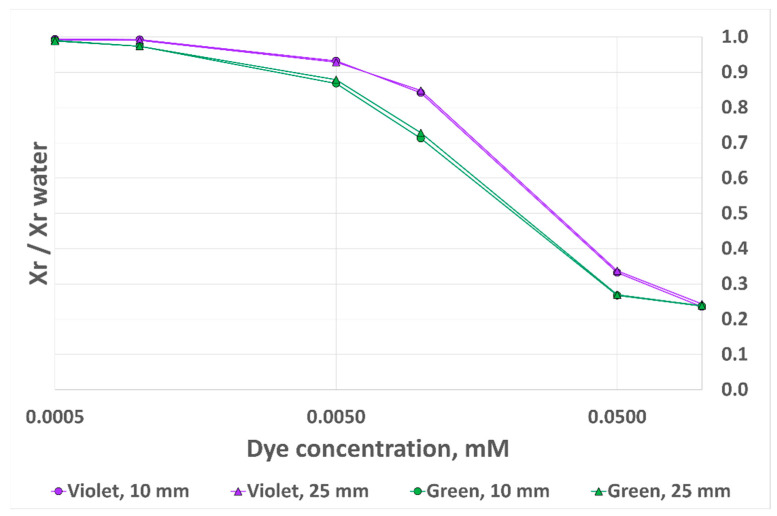
RGB sensor output signal variation with dye concentration for two dyes and two analyte heights, 10 mm OP prototype, alternative output metrics.

The result obtained with this updated metric is presented in Figure 12. As can be observed, the discrepancies between the two curves for each dye are significantly decreased. The error is now 2.2% for MG and 3.3% for CV. However, we observe a lower sensitivity at low dye concentrations, as well as a lower saturation threshold at higher dye concentrations. We therefore believe that the best way to eliminate the impact of analyte height is to ensure that the cuvette filling height is above the previously demonstrated threshold: 12 mm for the 10 mm OP cuvette and 28 mm for the 40 mm OP cuvette.

**Figure 13:**
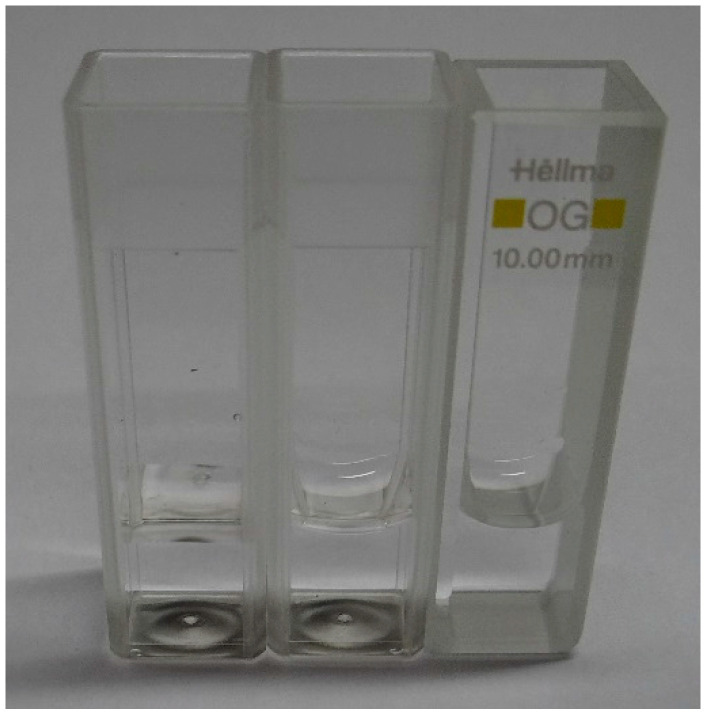
Water to cuvette meniscus for a new PMMA cuvette (**left**), cleaned PMMA cuvette (**middle**) and optical glass cuvette (**right**).

Finally, we performed the last experiment, during which we placed the exact same volume of water (1 mL), using an accurate laboratory pipette, in several types of cuvettes. When using a plastic (PMMA) cuvette, no meniscus was observed. When using optical glass cuvettes, a meniscus was clearly observable (Figure 13). We could also obtain a meniscus in the plastic cuvette after cleaning it with isopropanol. Although plastic cuvettes are single-use and not intended for cleaning, we compared the RGB sensor measurements obtained with new and cleaned cuvettes to exclude the impact of optical property mismatches between plastic and glass. We observed a significant difference between the two plastic cuvettes, between 15% and 17% according to the RGB channel. This result constitutes an additional reason so as not to use this measurement system at analyte volumes below the identified threshold.

### 3.2. Impact of Deposits and Floating Particles

The objective in this section is to assess the effectiveness of the prototype in performing a quick chemical analysis assay in which the reagent is in the form of a powdery solid and is directly poured into a cuvette prefilled with the analyte. In our case study, the reagent was MG dye-loaded MIP imprinted for the detection of Methoxphenidine (MXP) [21]. Based on preliminary experiments, we observed that the powdery reagent, after brief manual shaking and a rest period of a few minutes, often partially stayed entrapped at the water–air interface, while most of it accumulated at the bottom of the cuvette. In order to make the sensing system sensitive only to the dye released in the solution, we studied the impact of floating particles and deposits on the sensor output signal. We anticipated that the floating particles would have an impact only if the analyte volume height in the cuvette was below the aforementioned threshold, i.e., if the sensor measured a significant part of the light reflected at the analyte-to-air interface. Concerning the reflection that occurred at the bottom of the cuvette, we did not take it into account previously, as it was constant and independent of the analyte volume in the cuvette.

**Figure 14:**
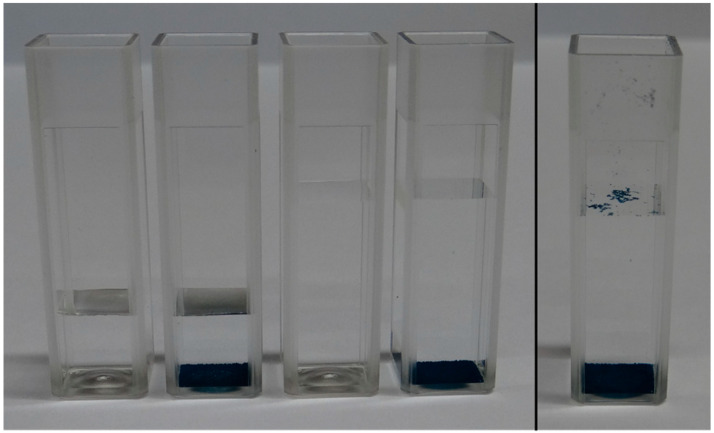
Samples used for the deposit and floating particle measurements: (**left**) without floating particles, (**right**) with added floating particles.

**Figure 15:**
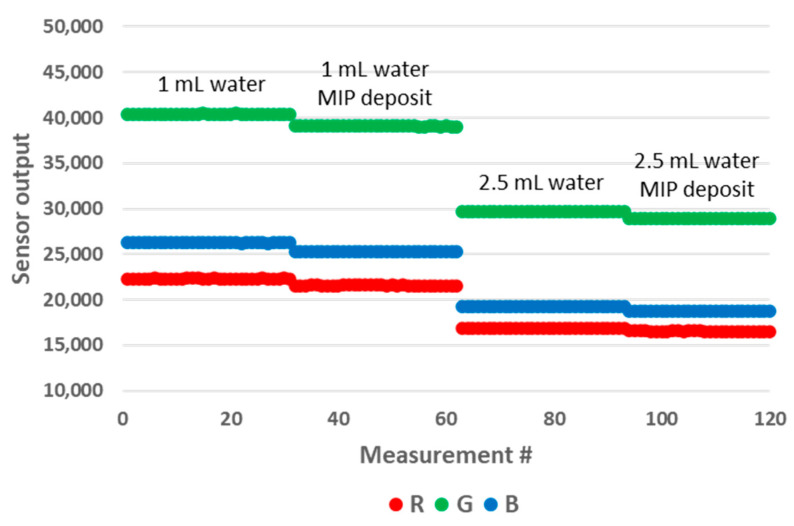
Impact of the deposit on the RGB sensor output for water volumes of 1 mL (**left part**) and 2.5 mL (**right part**) in the 10 mm OP prototype.

**Figure 16:**
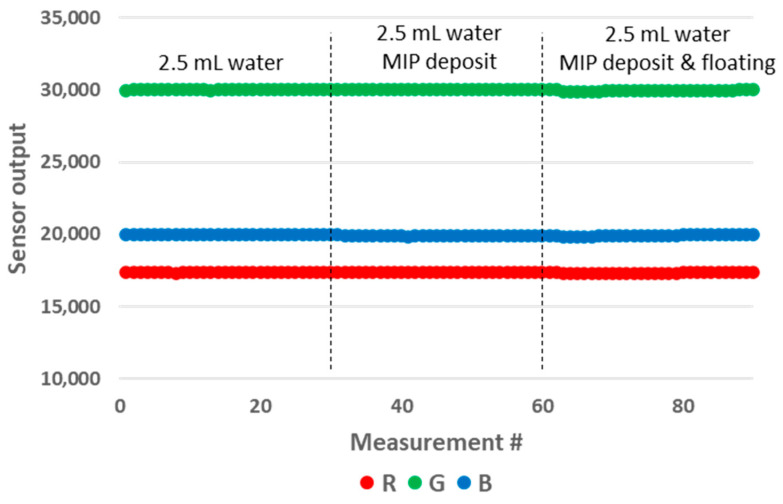
Impact of the deposit and floating particles on the RGB sensor output for a water volume of 2.5 mL in the modified 10 mm OP prototype.

The samples used for these experiments are presented in Figure 14. We prepared samples of 1 mL and 2.5 mL of water in 10 mm OP PMMA cuvettes, with and without the addition of 4 mg of MIP. After the addition of MIP, the cuvette was shaken manually for five seconds, after which a resting period before decantation was included for fifteen minutes. We observed no floating particles with the MIP batch that we used that day (Figure 14, left), while we could not avoid floating particles with the samples prepared previously with another batch of MIP. We believe that the effective size of MIP aggregates might play a role in this matter, as this modifies their wettability. The results of the measurements are presented in Figure 15. The MIP deposit at the bottom of the cuvette had a significant impact on the sensor output. Therefore, this prototype configuration is not suitable for performing these types of tests. In an attempt to mitigate the impact of the deposit, we modified the design of the 3D printed enclosure. In the modified version, the height of the LED/RGB sensor was increased to 14 mm above the bottom of the cuvette. The results obtained with this new configuration are presented in Figure 16. We could not observe any sensor output variation with the presence of the deposit. In order to measure the impact of floating particles, we added about 1 mg of MIP to the cuvette, without subsequent shaking (Figure 14, right). In this case, no output signal variation was observed. We performed the measurements in the updated prototype with a 2.5 mL water volume so that the water height was above the LED/RGB sensor height. In this case, the water-to-air interface was located 11 mm above the LED/RGB sensor centre line. This is well above the previously identified threshold of 5.5 mm, above which the sensor output becomes independent of the analyte level. In a similar manner, it appears that there is also a minimal sensor/RGB sensor height above which the presence of a deposit at the bottom of the cuvette has no impact. This minimal height was not studied in the present work, as it is likely application-dependant (material of the deposit, deposit volume, etc.).

## 4. Conclusions

Although very promising for the development of portable and affordable colorimetric analysers, LED-based systems champion a straightforward design with no control of the light path. In this paper, we analysed how inaccurate sample volume measurements, reduced analyte volumes and the presence of deposits and floating particles in the cuvette impact the measurement system’s response. To the best of our knowledge, this is the first time that such a study has been performed. For this purpose, we implemented a portable colorimetric analyser based on commercial spectrophotometer cuvettes, 3D printed parts and a limited number of readily available electronic components. We demonstrated that there is an analyte height above the LED/RGB sensor above which the output signal of the RGB sensor stabilises: about 5.5 mm for the 10 mm OP prototype and about 21 mm for the 40 mm OP prototype. We also demonstrated that there is a sensor height threshold above which the presence of deposits at the bottom of the cuvette has no effect. Based on these results, we demonstrated how the prototype could be used to implement quick and cost-effective dye-displacement assays. While we showed that using alternative output metrics could significantly reduce the impact of analyte volume on the measurement, they should only be used in specific cases, as they reduce the system’s sensitivity and operational range. Furthermore, these metrics would not help in the case of floating particles. In conclusion, provided that preliminary optical characterisation is carefully conducted and experimental conditions such as the minimal analyte volume are well defined, LED-based systems can be used advantageously in a wide range of applications, both in the field and in the lab.

## Data Availability

Data is contained within the article.
